# Osseous Metaplasia of the Cervix: A Rare Transformation Can Mimic a Tumor—Literature Review

**DOI:** 10.1155/2018/1392975

**Published:** 2018-10-31

**Authors:** Ameerah Alsaqobi, Nabeel Al-Brahim

**Affiliations:** ^1^Sixth Year Medical Student at Kuwait University, Kuwait; ^2^Department of Pathology, Farwaniya Hospital, Kuwait

## Abstract

**Background:**

The transformation of nonosseous soft tissue into bone is known as osseous metaplasia (OM). This condition most commonly affects the musculoskeletal and central nervous systems and it is a well-known phenomenon in different soft tissue organs. Rarely, OM can affect the uterus, which can extend into the cervix. OM affecting the cervix alone is a more rare condition that has multiple different clinical presentations. The presentation can be similar to that of a tumor in extremely rare cases.

**Case Summary:**

A 23-year-old nulligravida was complaining of irregular vaginal bleeding for one-month duration. Speculum examination revealed a foul-smelling bloody purulent discharge, tender cervix, and a brownish growth located at the posterior cervical lip. A punch biopsy of the growth was performed. Histological examination of the tissue revealed multiple bone fragments with necrosis and an inflammatory exudate. Because of the unusual findings, a repeat biopsy was performed. The biopsy yielded the same findings, which confirmed the diagnosis of osseous metaplasia of the cervix.

**Conclusion:**

Although osseous metaplasia is a known phenomenon in different soft tissues, it is extremely rare in the uterine cervix and can mimic malignancy. Therefore, clinicians should be aware of it.

## 1. Introduction

Osseous metaplasia (OM) is the benign transformation of nonosseous soft tissue into bone. The musculoskeletal and central nervous systems are the most commonly affected tissues by this condition [1]. It is a well-known phenomenon in different soft tissue organs including the colon, thyroid, and adrenal glands [2–4]. However, it is very rarely reported in the uterine cervix [5–12]. OM of the cervix can present with different symptoms including: dyspareunia, pelvic pain, leukorrhea, menstrual irregularities, and postcoital bleeding.

Rarely, OM can mimic a tumor formation. In this paper, our aim is to report a case of cervical OM mimicking a cervical tumor. In addition, we include a literature review of the previously reported cases.

## 2. Case Description

A 23-year-old presented with irregular vaginal bleeding for one-month duration. The patient was also complaining of a foul-smelling discharge, abdominal pain and unusual sensation of a mass in her vagina. The patient was never pregnant before. She is medically free. The patient reported using some herbal treatment topically on her genitalia. On examination of the external genitalia, the vulva and perineum were normal. Bimanual exam revealed that the uterus was anteverted and normal in size. The adnexa and pouch of Douglas were normal as well. Speculum examination, however, revealed a foul-smelling bloody purulent discharge, and a brownish growth located at the posterior cervical lip. In addition, the cervix was tender on palpation. Given that a vaginal bleeding can be the first symptom of a cervical malignancy together with the finding of a mass on examination the case was suspicious for a cervical malignancy.

**Figure 1:**
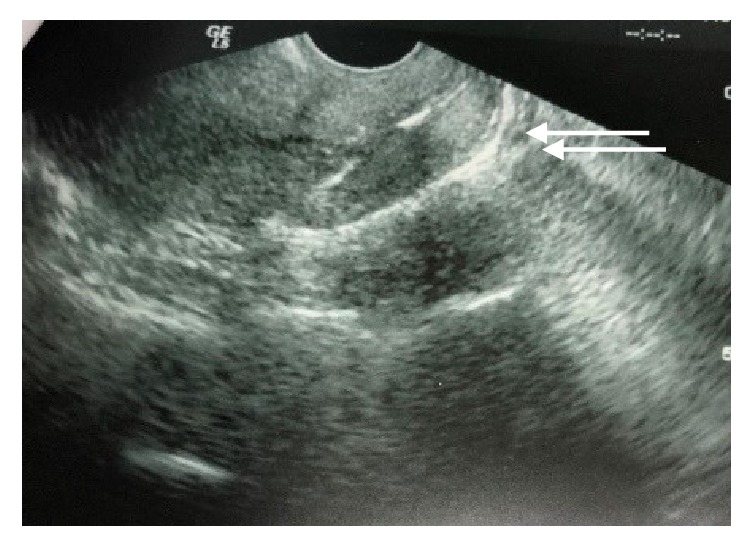
Ultrasound showing a hyperechoic cervix.

A transvaginal ultrasound was done for the patient. The ultrasound showed a normal sized anteverted uterus with normal echogenicity. The endometrium was well defined but appeared thin. The ovaries were normal in size and showed multiple small follicles. The adnexa were normal bilaterally. The pouch of Douglas was free, all consistent with the examination findings. The cervix, however, was hyperechoic Figure 1. A punch biopsy of the growth was taken for histological examination. The specimen consisted of multiple fragments of brown soft tissue measuring 1 cm in aggregate. The tissue histological section demonstrated multiple bone fragments with necrosis and an inflammatory exudate.

**Figure 2:**
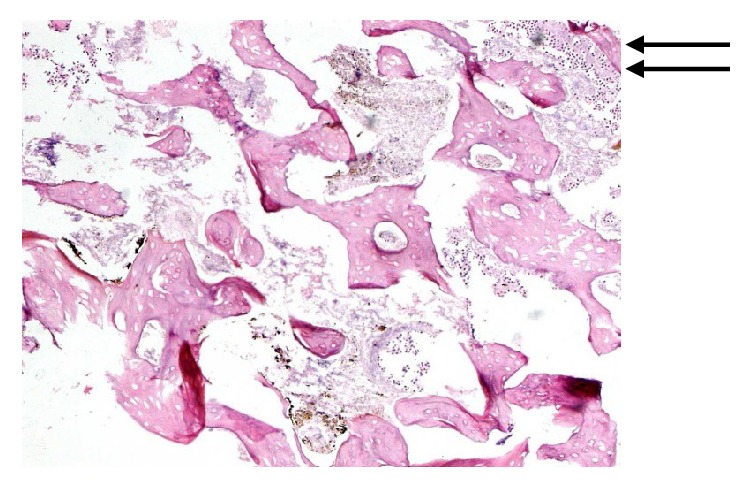
Histological section demonstrating mature bone with inflammatory exudate.

Additionally, benign squamous epithelium was seen Figure 2. The metabolic profile of the patient, including calcium levels was normal. Due to the very unusual findings a repeat biopsy was requested, which again yielded the same results, confirming the diagnosis of OM of the cervix. The patient was discharged against medical advice. She was lost to follow up with no intervention performed.

## 3. Discussion

Metaplasia is the reversible substitution of one type of fully differentiated cell for another within a given tissue [13]. The most common type is the conversion from squamous to glandular cells and vice versa [14]. Heterotrophic ossification, or OM, is transformation of nonosseous soft tissue into bone.

There are two forms of OM, a congenital and an acquired form. In both situations, the ossification process is initiated by a local osteogenic factor, which stimulates osteoblasts to differentiate and synthesize ground substance and collagen [15]. The acquired form of OM is most frequently seen with either musculoskeletal trauma, or central nervous system injury [1, 16]. OM occurring in the cervix is a very rare entity and it can sometimes be associated with OM of the uterus.

OM of the cervix can be asymptomatic or clinically present with leukorrhea, pelvic pain, vaginal bleeding, menstrual irregularities, infertility, or dyspareunia, Table 1. Most of the previously reported cases had a history of an abortion or a chronic condition of the cervix causing injury, such as an infection or intraepithelial neoplasia. Out of the 8 cases in the literature, three presented with infertility and two with intermenstrual bleeding. Two other women presented with dyspareunia and only one with a vaginal discharge. No previously reported case had a presentation of a tumor as in our patient. The diagnosis is suggested by an ultrasound, which shows an echogenic focus. Confirmation can then be done with a biopsy which shows mature bone. Rarely, OM of the cervix can mimic malignancy as in our case. A biopsy can help in making the correct diagnosis.

The exact mechanism of OM of the cervix is not known although several have been proposed. It has been suggested that inflammation and trauma leading to chronic tissue injury stimulate undifferentiated stromal mesenchymal cells to undergo OM [17]. In case of OM of the cervix, inflammation and trauma are in the form of cervical infections, cervical intraepithelial neoplasia, or cervical biopsy [7]. In our patient, the findings of a purulent discharge and tender cervix indicate ongoing cervical inflammation. It is a possibility that this was caused by her use of topical herbal treatments. We hypothesize that this treatment, which contains unknown chemicals, caused irritation and inflammation of her cervix leading to OM. Moreover, the possibility of an infection causing chronic inflammation and leading to OM is very likely in our patient since a purulent cervical discharge is characteristic of gonorrheal and chlamydial cervicitis [18]. Unfortunately, testing for these organisms was not available for the present case. Another suggested mechanism is the retention and direct implantation of fetal bone in the uterus after an abortion, leading to OM of the uterus that may or may not extend to the cervix. However, this theory was disputed for several reasons. Firstly, it has been shown that the osseous tissue has the same DNA as the patient, excluding a fetal origin [19]. Secondly, no areas of endochondral ossification are seen in the histologic examination of the transformed tissue as it would be expected in developing fetal bone [5].

Finally, some women develop uterine OM after abortions occurring before 13 weeks of gestation, when fetal bone starts developing [20, 21]. Nonetheless, it is worth noting that the retention of fetal tissue can possibly lead to OM of the uterus through chronic inflammation. This inflammation then acts as a stimulator of proliferation of mesenchymal cells that have inherent property of metaplasia and can differentiate into chondroblasts or osteoblasts [22]. In the present case, OM is limited to the cervix as evidenced by the normal echogenicity of the uterus on ultrasound. However, more invasive tests, such as hysteroscopy, were not performed.

This condition is distinct from metastatic and dystrophic calcification. Metastatic calcification occurs when the calcium-phosphorous levels are elevated, and the calcifications involve normal tissues. Dystrophic calcification, however, occurs in the presence of normal metabolism in damaged or devitalized tissues [23]. Calcification can mimic the appearance of bone on ultrasonography; therefore, excluding disorders leading to either metastatic or dystrophic calcification in cases of OM of the cervix is paramount. In tissue sections, OM can be easily distinguished from dystrophic calcifications by the presence of osteoblasts [24]. It is worth noting that most of the cases of OM of the uterus reported did not have any evidence of hypercalcemia or conditions leading to hypercalcemia, as seen with our patient [25].

## 4. Conclusion

OM of the cervix is not a well-recognized condition due to its rare occurrence. This condition is benign, and treatment is available with resection. Therefore, it is important to recognize its occurrence to avoid misdiagnosis as a malignant tumor, such as a malignant mesodermal tumor.

## Figures and Tables

**Table 1:** Literature review.

Case	Study	Patient age	Clinical presentation	Related medical history	Imaging findings	Associated OM of the uterus
1	Bhatia and Hoshiko, 1982 [5]	24	Hypomenorrhea, and intermenstrual bleeding	One elective abortion	Pelvic x-ray showed foci of calcifications Pelvic CT showed an amorphous irregularly defined, lobulated, soft tissue density in the pelvis with central areas of calcification Transvaginal ultrasound could not be inserted beyond cervical canal because of hard resistance	Yes

2	Bedaiwy et al., 2001 [6]	31	Passing small pieces of white hard material and dyspareunia	Two abortions, one elective and another spontaneous. CIN stage III treated with LEEP	Transvaginal ultrasound revealed a dense echogenic area in the cervix and the lower uterine segment consistent with calcification	No

3	Sabatini et al., 2001 [7]	22	Postcoital bleeding and dyspareunia	One previous elective abortion and CIN stage II which was treated	NA	No

4	Cicinelli et al., 2005 [8]	41	Pelvic pain, dysmenorrhea and primary infertility	Current endocervctis with group B strep	NA	No

5	Polat et al., 2011 [9]	31	Infertility	Two previous elective abortions	Transvaginal ultrasound examination showed a highly echogenic focus that extended from the cervical region through the posterior wall of the endometrium.	Yes

6	Mandato et al., 2012 [10]	19	Vaginal discharge	One spontaneous abortion, HIV and HPV infections, and recurrent vaginitis	NA	No

7	Giannella et al., 2014 [11]	25	Hypomenorrhea, intermenstrual bleeding, and pelvic pain	Underwent LEEP 12 months ago for high grade CIN	Transvaginal ultrasound showed an image compatible with hematometra	No

8	Elkattan et al., 2015 [12]	30	Infertility, postcoital bleeding, and dyspareunia	NA	NA	No

9	Alsaqobi and Al- Brahim, 2018 (the present study)	23	Irregular vaginal bleeding, vaginal discharge, abdominal pain and an unusual sensation of a mass	NA	Transvaginal ultrasound showed a hyperechoic cervix	No

NA: not available; CIN: cervical intraepithelial neoplasia; LEEP: loop electrosurgical excision procedure; HIV: human immunodeficiency virus; HPV: human papilloma virus.

## Data Availability

No data were used to support this study.
